# Efficacy of Combining Multiple Electromagnetic Navigation Bronchoscopy Modalities for Diagnosing Lung Nodules

**DOI:** 10.3390/jcm11247341

**Published:** 2022-12-10

**Authors:** Ju Yeun Song, Sun Hye Shin, Hongseok Yoo, Byeong-Ho Jeong, Sang-Won Um, Hojoong Kim, O Jung Kwon, Kyungjong Lee

**Affiliations:** Division of Pulmonary and Critical Care Medicine, Department of Medicine, Samsung Medical Center, School of Medicine, Sungkyunkwan University, 81 Irwon-ro, Gangnam-gu, Seoul 06351, Republic of Korea

**Keywords:** electromagnetic navigation bronchoscopy, radial endobronchial ultrasound-guided transbronchial biopsy, lung nodule

## Abstract

Electromagnetic navigation bronchoscopy (ENB) is one of the non-invasive methods used for lung nodule biopsy. We evaluated the efficacy of combining radial endobronchial ultrasound (R-EBUS)-guided transbronchial lung biopsy (TBLB) with ENB-guided TBLB or transbronchial needle aspiration (TBNA) for diagnosing lung nodules. Forty patients with a lung nodule underwent ENB-TBLB or TBNA, followed by R-EBUS-TBLB if available. The final diagnosis was benign or malignant, depending on the surgical pathology or 24-month follow-up computed tomography (CT). We compared the sensitivity, negative predictive value, and accuracy between combinations of procedures. The mean nodule size was 21.65 mm, and 60.0% of the nodules were solid. The bronchus was within the nodule in 67.5% and 65.0% of cases examined using CT and R-EBUS, respectively. The accuracies of ENB-TBLB alone, ENB-TBLB/TBNA, and R-EBUS-TBLB plus ENB-TBLB/TBNA were 74.4%, 82.5%, and 90.0%, respectively. The sensitivity levels of the aforementioned procedures were 69.8%, 78.8%, and 87.9%, respectively. Among 21 patients who underwent both ENB-TBLB and R-EBUS-TBLB, the latter revealed malignant cells in three of nine patients (33.3%) with benign ENB-TBLB results. Combined ENB-TBLB/TBNA and R-EBUS-TBLB had increased sensitivity and diagnostic accuracy for lung nodules. ENB and R-EBUS are complementary; using both modalities improves the sensitivity and accuracy of lung nodule diagnoses.

## 1. Introduction

The diagnosis of lung nodules found incidentally on chest computed tomography (CT) can be challenging for physicians because the nodules are frequently located at the periphery of the lungs (i.e., beyond the reach of conventional bronchoscopy) [1]. Physicians must decide whether or not to recommend surgical resection for lung cancer patients. The traditional non-invasive approach to lung nodule biopsy is CT-guided transthoracic needle aspiration (TTNA). Although a meta-analysis indicated that this technique has good diagnostic performance (i.e., 92% accuracy) [2], TTNA has the potential for serious complications such as pneumothorax, which may require chest tube insertion, massive hemoptysis, and, occasionally, air embolism [3]. These complications may occur in patients with severe emphysema or a lung nodule with an air bronchus sign. As an alternative to TTNA, radial endobronchial ultrasound (R-EBUS)-guided transbronchial lung biopsy (TBLB) has been performed to diagnose lung nodules inaccessible via TTNA or appearing in patients at high risk of serious TTNA-associated complications [4]. A conventional bronchoscope with a diameter of 6 mm can only reach the subsegmental bronchus, and is therefore not suitable for sampling peripheral lung nodules. In contrast, a thin bronchoscope (4 mm diameter) in combination with R-EBUS can be used to obtain lung tissue in peripheral lung nodules. A meta-analysis reported that the diagnostic performance of R-EBUS was 70.6% (95% confidence interval: 68.0–73.1%) [5]. Electromagnetic navigation bronchoscopy (ENB) is another tool used for diagnosing lung nodules located in the periphery of the lungs [6]. In ENB, an electromagnetic generator with sensors enables a real-time biopsy of lung nodules under the guidance of computer software loaded with the patient’s CT images. In a recent study of 1092 samples, the 12-month diagnostic yield of ENB-guided TBLB was 73% [7]. In general, a bronchoscopic biopsy guided using R-EBUS or ENB appears to have a higher diagnostic yield compared with conventional bronchoscopic biopsy but a lower diagnostic yield compared with TTNA [8].

In this study, we evaluated the diagnostic performance of navigation bronchoscopy (ENB-TBLB plus transbronchial needle aspiration (TBNA)), and investigated whether or not R-EBUS could improve the diagnostic yield of ENB-guided TBLB/TBNA for lung nodules.

## 2. Materials and Methods

### 2.1. Study Subjects and Baseline Characteristics

Data collected in 2019 at the Samsung Medical Center (Seoul, South Korea) were retrospectively analyzed. Chest CT scans were examined to determine the characteristics of pulmonary nodules. We evaluated nodule size (short and long diameter), type (solid or part solid), and location, and the bronchus sign, on CT scans. The study protocol was approved by our Institutional Review Board (IRB no. 2022-10-018), and we were permitted to review and publish information acquired from patient records. The requirement for informed consent was waived because the patient information was de-identified and anonymized prior to the analysis.

### 2.2. Electromagnetic Navigation-Guided Bronchoscopy

Electromagnetic navigation-guided bronchoscopy (ENB) was performed in two steps. Prior to bronchoscopy, planning was conducted using software. Before the procedure, chest CT images were uploaded to the navigation system to construct a virtual image, to guide the physician to the lung nodules. The uploaded images were processed and the target nodules were shown on a screen during the procedure. The operator delineated the target lesion and mapped a path from the trachea to the nearest bronchus to approach the target. After the planning stage, a bronchoscope was introduced into the trachea and magnetic forceps were inserted into the inlet of the bronchus. After matching the chest CT scans to the patient’s bronchus, a pair of magnetic forceps or a needle was advanced to the target lesion according under navigation guidance. When the device reached the target lesion according to real-time virtual images, the target nodule was sampled via the magnetic forceps or needle.

### 2.3. Radial EBUS-Guided Biopsy

After completing the electromagnetic navigation-guided lung biopsy for a lung nodule, R-EBUS-guided biopsy was performed if the patients were able to tolerate another procedure under conscious sedation (in accordance with the bronchoscopist’s decision). The R-EBUS probe (1.4 mm, 20 MHz, UM S20-17S; Olympus, Tokyo, Japan) was inserted through the working channel of the thin bronchoscope. Once the probe reached the lung nodule, transbronchial biopsy was performed with 1.8 mm biopsy forceps.

### 2.4. Definitions of Diagnostic Standards

The final diagnosis was of a benign or malignant nodule, based on the cytological results of the ENB-TBLB or ENB, R-EBUS-guided TBLB, or surgery. If the ENB or R-EBUS biopsy results were indeterminate and the nodule was highly likely to be malignant, it was surgically resected or clinically followed via surveillance CT for at least 6 months. Inflammation or fibrosis was confirmed, i.e., the nodule was considered benign, when the nodule size decreased during surveillance or was stable for >24 months.

### 2.5. Statistical Analysis

Data are reported as numbers with percentages for categorical variables, and as median with interquartile range (IQR) or mean ± standard deviation for continuous variables. The specificity, sensitivity (Sn), negative predictive value, positive predictive value, and accuracy were calculated for each diagnostic modality. All analyses were performed using R software (version 3.2.2; R Foundation for Statistical Computing, Vienna, Austria).

## 3. Results

We retrospectively analyzed data from a total of 40 patients. The mean patient age was 67.45 years and 52.5% of the patients were female. The average procedure duration from the insertion of the bronchoscopy to the end of the ENB-TBLB or aspiration was 24.35 min. The average time between insertion of the radial probe and removal of the bronchoscope was 7.86 min. The mean doses of midazolam and fentanyl were 4.6 mg and 61.75 μg, respectively.

The mean nodule size was 21.65 mm in long diameter and 16.23 mm in short diameter, and 60.0% of the nodules were solid. The nodules were most frequently located in the LUL (27.5%), followed by the RUL (25.0%), RLL (25.0%), LLL (15.0%), and RML (7.5%). The bronchus sign on chest CT was “within the nodule” in 67.5% of patients and “adjacent to the nodule” in 27.5%. In terms of R-EBUS, the radial probe was located within the nodule in 65% of patients and adjacent to the nodule in 25%, although the nodule was invisible in 10.0% of cases. Among the lung nodules in all 40 enrolled patients, TBLB was available in 39 cases and unavailable in 1, with the latter being evaluated by ENB-TBNA (aspiration cytology). For ENB-TBLB or aspiration, adenocarcinoma was the most common diagnosis (46.2%) and 38.5% of the patients were negative for malignancy. Baseline demographics among the ENB-TBLB, ENB (aspiration cytology), and R-EBUS-guided TBLB are presented in Table 1.

Among the 40 patients, 39 were able to undergo ENB-TBLB. ENB (aspiration) was performed in 17 patients. R-EBUS-guided TBLB was performed in 21 patients. The lung nodule procedures are listed in Table 2. Malignancy was diagnosed in 59.0%, 52.9%, and 57.1% of patients who underwent ENB-TBLB, ENB (aspiration cytology), and R-EBUS-guided TBLB, respectively.

The diagnostic yield for malignancy was 79% in patients who underwent ENB and 71% in those who underwent R-EBUS-guided lung biopsy. However, the diagnostic yield increased to 88% when ENB and R-EBUS-guided TBLB were combined. We named the procedures TBLB1 (ENB-guided lung biopsy only), TBLB2 (ENB-guided lung biopsy plus needle aspiration), and TBLB3 (ENB-guided lung biopsy and aspiration plus R-EBUS-guided lung biopsy).

**Figure 1:**
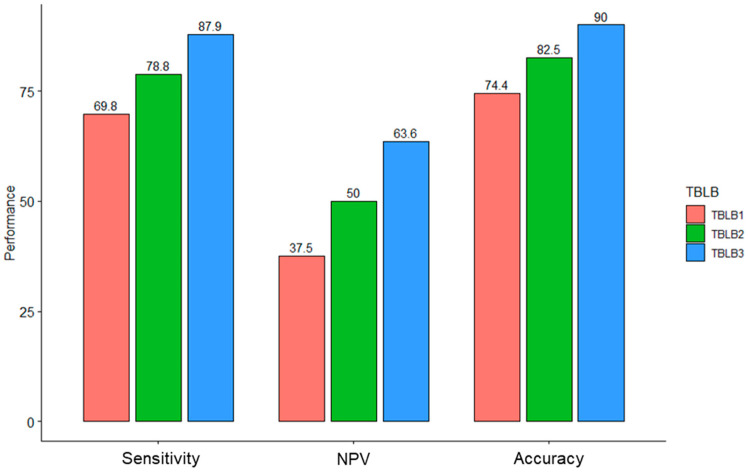
Performance of ENB and R-EBUS-guided TBLB. Diagnostic accuracy was boosted after adding another modality. NPV, negative predictive value; TBLB1, ENB-guided lung biopsy only; TBLB2, ENB-guided lung biopsy plus needle aspiration; TBLB3, ENB-guided lung biopsy and aspiration plus R-EBUS-guided lung biopsy.

The sensitivity was 69.8%, 78.8%, and 87.9% for TBLB1, TBLB2, and TBLB3, respectively. The details are given in Figure 1. The accuracy of ENB-TBLB was 74.4%, and that for ENB-TBLB with ENB-guided needle aspiration cytology was 82.5%. With the addition of R-EBUS-guided TBLB, accuracy rose to 90.0%. In 21 cases in which ENB and R-EBUS were performed together, R-EBUS enabled a diagnosis of malignancy in 33% of the patients with non-diagnostic results from ENB. In contrast, ENB identified additional malignant nodules in three cases for which R-EBUS-guided TBLB produced negative results. Diagnostic accuracy was also assessed according to the average diameter size of the enrolled pulmonary nodules (Table 3).

## 4. Discussion

We investigated the efficacy of combining ENB with R-EBUS-guided TBLB for diagnosing suspicious malignant lung nodules in 40 patients. The overall diagnostic accuracy increased from 82.5% to 90% after adding the R-EBUS results to the ENB-TBLB or aspiration data. Both R-EBUS and ENB are useful modalities for peripheral lung nodule diagnosis [9]. R-EBUS, which is a cost-effective procedure developed for the diagnosis of peripheral lung nodules, had a good success rate. A meta-analysis of R-EBUS [10] collected data from 7601 patients across 51 studies; the sensitivity of R-EBUS was 0.72, although the data had significant heterogeneity (I^2^ = 76%). Even though this success rate is high compared with flexible bronchoscopy, which has a yield of 30–60%, the data for R-EBUS-guided TBLB had high heterogeneity, suggesting that the success of this procedure might be bronchoscopist-dependent [11]. In contrast to EBUS-guided needle aspiration for mediastinal lymph nodes, which uses a convex probe and enables real-time guidance, the confirmation of target lesions during biopsy with forceps in R-EBUS is indirect, as it follows the detection of lung lesions with a radial probe.

ENB was developed to enhance diagnostic accuracy for peripheral lesions. It includes magnetic navigation system guidance plus the real-time visualization of target lesions via virtual images obtained during biopsy. Today, the most frequently used ENB devices are the SuperDimension^TM^ navigation system (version 6; Medtronic, Minneapolis, MN, USA) and Spin Thoracic Navigation System (SYS-4230 K; Veran Medical, St. Louis, MO, USA) [12]. A prospective randomized controlled trial (RCT) compared the diagnostic yields of ENB, R-EBUS, and ENB with R-EBUS [13] in 120 patients. The authors found that ENB (59%) or R-EBUS (69%) alone were inferior to ENB combined with R-EBUS (88%). Therefore, R-EBUS combined with ENB-TBLB has enhanced diagnostic power, without increasing the rate of procedure-related complications.

In the large, multicenter NAVIGATE prospective cohort ENB study [7], 1157 patients from 29 centers were enrolled. About half of the patients (57.4%) underwent R-EBUS. Although the overall diagnostic yield was 73%, ENB alone (76.4%) had a higher diagnostic yield than ENB with R-EBUS (70.6%). Because the main purpose of the study was to evaluate ENB, R-EBUS was selectively used for nodules that were difficult to access. This may explain the lower diagnostic yield of ENB with R-EBUS. Guler et al. examined the diagnostic yields of ENB with or without R-EBUS-guided TBLB. In their study, 56 patients underwent ENB and 26 underwent simultaneous biopsies with R-EBUS following ENB. The diagnostic yields for ENB with R-EBUS and ENB alone were 73.07% and 71.42%, respectively [14]. All aforementioned studies were performed using SuperDimension and Bronchus software (SuperDimension, Inc., Plymouth, MN) for ENB. Another meta-analysis examined the additional advantage of R-EBUS (1.4 mm, 20 MHz, UM S20-17S; Olympus) in combination with ENB (SuperDimension). However, the results were not suitable for meta-analysis because of study heterogeneity and small sample sizes [15]. SuperDimension and Bronchus software were used to navigate to the target nodule, and an extended working channel (EWC) was lodged into the target lesion. After confirming the location of the target nodule in the system, a transbronchial lung biopsy was conducted via the EWC. Compared with the SuperDimension ENB system, a spin thoracic navigation system using sampling devices including brushes, needles, forceps, and electromagnetic sensors at the tip enabled superior virtual visualization for real-time biopsy [16]. However, most of the previously published data were collected using the SuperDimension navigation system. Thus, information regarding the performance of the spin thoracic navigation system in combination with R-EBUS is limited.

A multivariate analysis conducted as part of the NAVIGATE study showed that the use of fewer than three tools was related to a higher diagnostic yield (OR: 1.44, *p* = 0.04). The authors explained that this was likely related to lesion complexity, i.e., compared with simple lesions, complex ones were more likely to have been evaluated using at least tools [7]. The findings of the present study were consistent with the results of the previous RCT. Specifically, the diagnostic yield was superior when all possible modalities were used, such as needle aspiration cytology plus TBLB and ENB, along with R-EBUS-guided TBLB, rather than each modality alone.

There were several limitations to this study. First, it used a retrospective, single-center, single-arm design and the procedures were performed by one operator. This limitation prevents us from generalizing our results regarding the combination of ENB plus R-EBUS modalities to other populations. Another limitation is that additional modalities following ENB were selected according to the preference of the physician. However, we found a concordance rate of 75% between the ENB- and R-EBUS-guided procedures in our patient group. In non-concordant cases, malignancy was confirmed by R-EBUS in three of the nine negative cases via ENB, and three of the nine negative cases via R-EBUS.

## 5. Conclusions

This study suggests that ENB-TBNA, ENB-TBLB, and R-EBUS-guided TBLB have synergistic effects that could improve the diagnostic performance for suspicious peripheral lung nodules. The combination of ENB systems and R-EBUS may overcome the drawbacks associated with each modality. However, further prospective research is required to validate the results of this study.

## Figures and Tables

**Table 1:** Demographic and baseline characteristics.

	N (%) or Mean ± Standard Deviation			
Characteristics	Overall	ENB-TBLB	ENB-TBNA	Radial EBUS
No. of patients	40	39	17	21
Sex				
Female	21 (52.5%)	20 (51.3%)	8 (47.1%)	12 (57.1%)
Age, years	67.45 ± 9.67	67.6 ± 9.7	66.9 ± 11.0	66.7 ± 10.6
ENB, min	24.35 ± 9.46	24.6 ± 9.5	26.3 ± 7.9	24.4 ± 10.6
R-EBUS, min	7.86 ± 8.52	8.0 ± 8.6	7.2 ± 7.7	13.6 ± 7.2
Midazolam, mg	4.6 ± 1.24	4.6 ± 1.2	4.6 ± 1.4	4.8 ± 1.3
Fentanyl, μg	61.75 ± 20.86	62.3 ± 20.8	62.4 ± 23.3	62.9 ± 21.7
Nodule size, mm		19.2 ± 6.8	16.4 ± 5.2	20.4 ± 8.1
Nodule type				
Solid	24 (60.0%)	23 (59.0%)	13 (76.5%)	11 (52.4%)
Part solid	16 (40.0%)	16 (41.0%)	4 (23.5%)	10 (47.6%)
Nodule location				
RUL	10 (25.0%)	10 (25.6%)	5 (29.4%)	6 (28.6%)
RML	3 (7.5%)	3 (7.7%)	1 (5.9%)	3 (14.3%)
RLL	10 (25.0%)	9 (23.1%)	3 (17.6%)	5 (23.8%)
LUL	11 (27.5%)	11 (28.2%)	7 (41.2%)	3 (14.3%)
LLL	6 (15.0%)	6 (15.4%)	1 (5.9%)	4 (19.0%)
Bronchus sign on chest CT				
Invisible	2 (5.0%)	2 (5.1%)	2 (11.8%)	0 (0.0%)
Adjacent	11 (27.5%)	10 (25.6%)	7 (41.2%)	4 (19.0%)
Within	27 (67.5%)	27 (69.2%)	8 (47.1%)	17 (81.0%)
EBUS images				
Invisible	4 (10.0%)	4 (10.3%)	4 (23.5%)	2 (9.5%)
Adjacent	10 (25.0%)	9 (23.1%)	5 (29.4%)	3 (14.3%)
Within	26 (65.0%)	26 (66.7%)	8 (47.1%)	16 (76.2%)

ENB, electromagnetic navigation bronchoscopy; TBLB, transbronchial lung biopsy; TBNA, transbronchial needle aspiration, R-EBUS, radial endobronchial ultrasound.

**Table 2:** Diagnostic results of ENB and R-EBUS-guided TBLB.

ENB-TBLB (*n* = 39)	
Negative for malignancy	16 (41.0%)
Positive for malignancy	23 (59.0%)
ENB-TBNA (*n* = 17)	
Negative for malignancy	8 (47.1%)
Positive for malignancy	9 (52.9%)
R-EBUS-TBLB (*n* = 21)	
Negative for malignancy	9 (42.9%)
Positive for malignancy	12 (57.1%)

**Table 3:** Diagnostic performance in each modality according to the size.

Performance (%)	<1 cm (*n* = 2)	1–2 cm (*n* = 19)	2–3 cm (*n* = 18)	≥3 cm (*n* = 1)
TBLB1				
Sensitivity	0	62.5	86.7	0
NPV	Na	33.3	60.0	Na
Accuracy	0	68.4	88.9	0
TBLB2				
Sensitivity	100	75.0	86.7	0
NPV	100	42.9	60	Na
Accuracy	100	78.9	88.9	0
TBLB3				
Sensitivity	100	81.2	93.3	100
NPV	100	50.0	75.0	Na
Accuracy	100	84.2	94.4	100

NPV, negative predictive value; TBLB1, ENB-guided lung biopsy only; TBLB2, ENB-guided lung biopsy plus needle aspiration; TBLB3, ENB-guided lung biopsy and aspiration plus R-EBUS-guided lung biopsy.

## Data Availability

All data are included in this manuscript.
